# Evaluation of industrial ecology in the π-shaped curve area of China’s Yellow River based on the grey Lotka–Volterra model

**DOI:** 10.1038/s41598-023-46618-7

**Published:** 2023-11-04

**Authors:** Yifang Sun

**Affiliations:** Northwest University, Xi’an, 710127 Shaanxi Province China

**Keywords:** Environmental social sciences, Environmental economics

## Abstract

Industrial ecology (IE) is the process of constructing industrial-ecological systems (IES) with the realization of the mutual benefits for industrial system (IS) and ecological system (ES). Therefore, the IE level depends on the IES development and the relationship between IS and ES. This paper calculated the development index of ES and IS to account for IES development and adopted the grey Lotka–Volterra to analyze the relationship between IS and ES. Choosing the π-shaped curve area in the Yellow River basin of China as the study area, this paper analyzed its IE level and influence factors from 2005 to 2019. Findings include: (1) The development level of IES in the Area presented significant spatial differentiation and could be classified into three levels. (2) IS and ES remained in the stage of isolated development or mutual restriction. (3) IE was found to be positively correlated with scientific innovation, economic development, openness degree, pollution control, and industrial structure while negatively correlated with urbanization, resource consumption, and government influence.

## Introduction

While the industrial revolution has created unprecedented material wealth in human history, it has also posed severe challenges to the relationship between humans and nature due to its external diseconomies^1,2^. In this context, re-examining and adjusting the relationship between the ecological system (ES) and the economic system has become an urgent issue to be solved. The industrial system (IS) is the core of the economic system and the link between the ES and the economic system. It profoundly affects the utilization of resources and the output of economic activities and determines their impact on the environment^3^. How to ensure the rapid development of the industrial economy under the sustainable support of environmental carrying capacity has attracted global attention. Since the International Conference on Industrial Ecology (IE) was held in Sweden in 2005, the idea of IE has been highly valued by many governments and international organizations.

IE was first proposed in 1989 by Frosch and Gallopoulos, arguing that the traditional IS should be transformed into industrial-ecological complex systems through technological innovation, and this transformation process is called IE^4^. Existing research on IE can be distinguished into micro-scale and macro-scale in terms of research subjects. Specifically, micro-scale studies emphasize the economic and ecological enhancement of industrial activities through structural and organizational transformations within the IS^5–9^. With the realization that IES could not exist in isolation from the constraints and influences of the socio-economic environment, the connotation of IE gradually extends to the macro-scale, emphasizing the design of IS that develops in symbiosis with the ecological system (ES) and ultimately realizing the sustainable region development^10–12^. Despite the difference in understanding perspectives, both micro and macro studies agree that the core idea of IE is to recognize the ecological environment and industrial development as an organic whole. IE evaluations can generally be divided into three types. The first one is to use characteristic indices such as life cycle assessment^13^, material flow analysis^14^, eco-efficiency evaluation^15^, environmental accounting^16^, and emergy theory^17^ to characterize the IE level. The second one is to evaluate the IE level by constructing a comprehensive index system^18,19^. The third type is by constructing relationship models between industrial development and the ecological environment to characterize the IE level^3,20,21^.

The IS is formed by the coexistence and compatible coupling of several industries of the same attributes^22^. Driven by external inducements and internal motivations, the IS experiences evolution from low level to high level, from immature to mature, and from simple to complex^23^. IS is the application of system science in the field of economics and is also the object of IE. Based on existing research^24–27^, this paper defined IE as the process of constructing industrial-ecological systems (IES) with the realization of the mutual benefits for IS and ES. Therefore, the IE level depends on the development level of the IES and the relationship between IS and ES.

Most of the existing studies on inter-system relationships used coordination degree models, which could be divided into static coordination models and dynamic coordination models^28^. The static coordination model includes the coupling coordination model^29^, membership functions^30^, deviation coefficient minimization model^31^, distance coordination model^32^, and data envelopment model^33^. The static coordination model assumes that the smaller the difference between the development level of the system and the ideal level, the more coordinated the system is. The shortcomings are that the ideal level is difficult to determine and ignores the interaction between the systems. The dynamic coordination model includes the elastic coefficient method^34^ and the system evolution method^35^. It assumes that the closer the evolution speed is between systems, the more coordinated the system is, overlooking the development level of the system itself.

The Lotka–Volterra (LV) model, initially proposed by Lotka^36^ and Volterra^37^, is often used to characterize the relationships between biological populations. For example, Lauro et al.^38^ used the LV model to describe how cooperation within a microbial system is impacted by periodic fluctuations in environmental parameters on key populations of microorganisms. Szolnoki et al.^39^ reviewed zero-dimensional rock-paper-scissors models and the application of the LV model to study the symbiosis and competition in a structured population. The LV model is also applicable in other research fields. For example, Zhang et al.^40^ discussed the effects of the smart growth plan on population, and economy-environment-society based on LV equations, and Xia et al.^41^ constructed a symbiosis evolution model of science communication ecosystem between scientists and social media platforms with the help of LV model. The nonlinear least-square method used to estimate the model's coefficients is based on the model's initial values and could lead to errors easily^42^. To solve this problem, Wu et al.^43^ proposed a grey LV (GLV) model with accuracy verified^44–46^.

**Figure 1:**
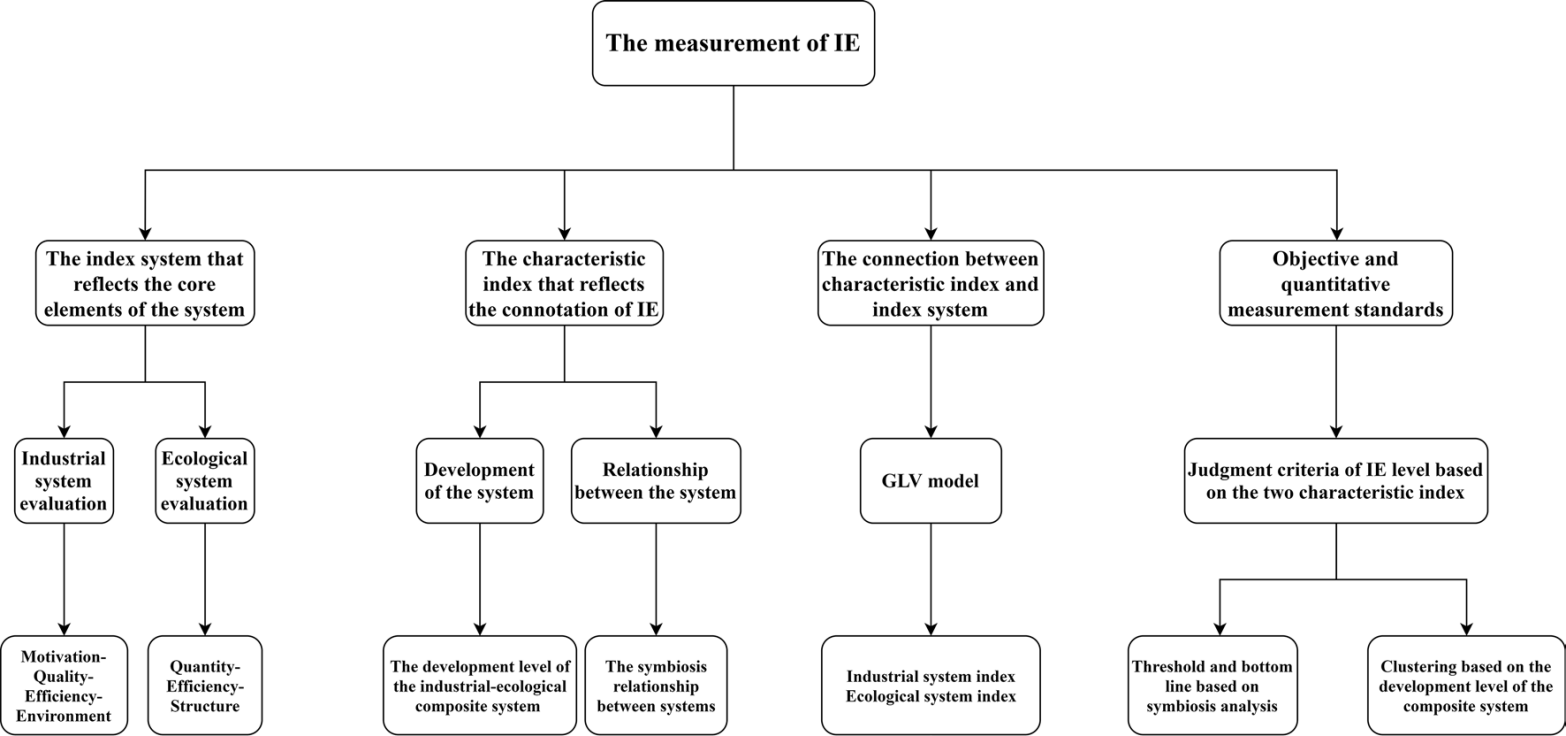
The Goal and Means Tree for measuring IE level.

The Goal and Means Tree was used to construct the logic for measuring IE (Fig. 1). According to the definition given in this paper, the IE level depends on the development level of IES and the relationship between IS and ES. This paper used the ES and IS evaluation framework proposed by Sun and Wang^1,47^ to account for the IES development and adopted the grey Lotka–Volterra to analyze the relationship between IS and ES. To analyze the influencing mechanisms of IE, this paper used the Oprobit model to identify IE influencing factors.

## Methods and data

### Study area and data sources

**Figure 2:**
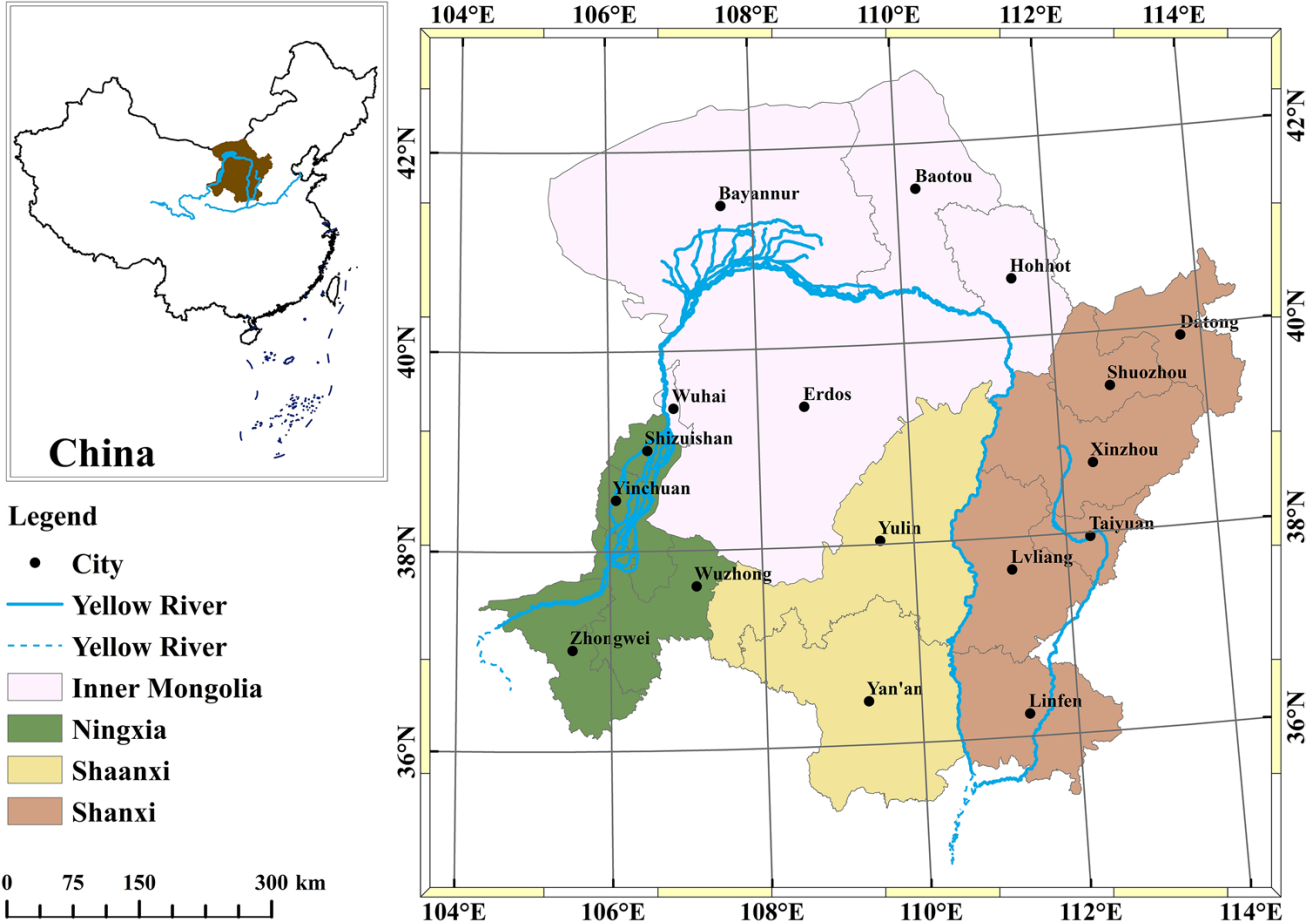
The location of the π-shaped Curve Area in the Yellow River basin.

As one of the most significant growth poles in west China, the π-shaped Curve Area in the Yellow River basin is characterized by a single industrial structure and a fragile ecological environment. It is of theoretical and practical significance to select this area as the study area of IE^48^. The π-shaped Curve Area included part of the upper reaches and the whole middle reaches of the Yellow River Basin, forming a “π-shaped” Curve Area^49^. Based on the naturally formed basin, this paper defined the π-shaped Curve Area based on the naturally formed watershed and the existing studies considering urban planning and administrative division integrity. The region includes 17 cities in Ningxia, Shaanxi, Inner Mongolia, and Shanxi provinces, including Shizuishan, Wuzhong, Yinchuan, Zhongwei, Hohhot, Baotou, Bayannur, Yan'an, Yulin, Ordos, Wuhai, Datong, Linfen, Taiyuan, Xinzhou, Shuozhou, and Lvliang^47^ (Fig. 2).

This paper studied the IE level in the Curve Area from 2005 to 2015. Data were obtained from the China Urban Statistical Yearbook^50^, China Urban Construction Statistical Yearbook^51^, China Forestry and Grassland Statistical Yearbook^52^, Shaanxi Statistical Yearbook^53^, Shanxi Statistical Yearbook^54^, Ningxia Statistical Yearbook^55^, Inner Mongolia Statistical Yearbook^56^, and the statistical yearbooks of 17 cities^57^.

### Theory foundation

**Figure 3:**
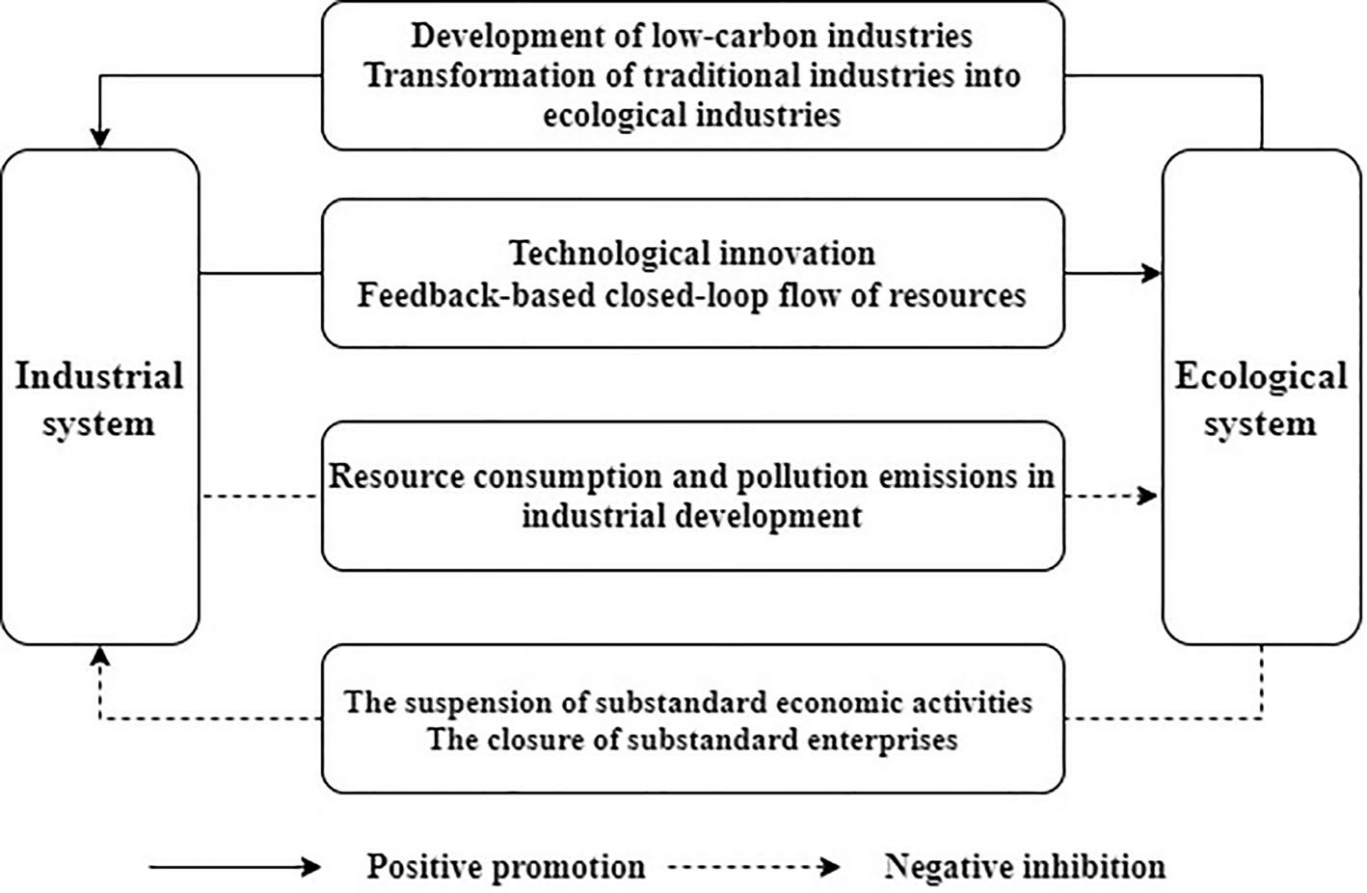
The relationship between ES and IS.

The interaction between IS and ES is a kind of functional coupling, a coupling of economic, ecological, and social benefits. This paper lists 4 typical interaction patterns here. (Fig. 3).

Type I: The development of the industrial economy can promote the improvement of the ecological environment, and the improvement of the ecological environment can promote the development of the industrial economy. When the intensity of industrial activities does not exceed the carrying capacity of the ecological environment, the IS development provides technology and funds for environmental protection that promotes ES development. At the same time, improving the ecological environment also provides the basis for further IS development.

Type II: The IS development accumulates material wealth while increasing people's ecological needs, thus promoting ES development. In the social context of advocating green production and environmental protection, high production and emission standards could lead to low profits for some traditional industries. Some enterprises that do not meet production standards even go bankrupt and close down, thus inhibiting the development of the regional industrial economy.

Type III: When the intensity of industrial activities exceeds the carrying threshold of the ecological environment, it will put pressure on the ES. If this situation continues, it may cause irreversible effects on the ecological environment, weakening the supply capacity for natural capital and limiting IS development. The IS and the ES compete with each other, forming a vicious circle.

Type IV: The ES is under pressure from industrial development, but through the transformation of traditional industries and the development of low-carbon industries, the industrial development efficiency is improved, which promotes the development of IS.

From the perspective of the causal relationship between ES and IS, environmental problems all originate from the anti-ecological process of industrial activities. Therefore, IE is to internalize ecological benefits in the process of industrial development so as to achieve a symbiosis between IS and ES. It is worth noting that ecological pressure is the inner thrust of the IS to change from the pursuit of quantitative growth to the pursuit of quality improvement. Meanwhile, the intensive development approach of the IS can optimize resource allocation, alleviate ecological pressure, and ultimately promote ecological improvement. Therefore, Type IV and Type II may be the transitional stage during the development from Type III to Type I. The competitive and cooperative relationship between the IS and ES is the inherent requirement for realizing regional IE.

The interaction between IS and ES is in line with the design of symbiosis theory. The concept of symbiosis was first proposed by German biologists Paracer and Ahmadjian^58^, which mainly describes the co-existence and synergistic development of different biological species. Symbiosis theory originated from ecology but has expanded beyond ecology and gradually into the social sciences and philosophy^59,60^. It is more of a methodology and epistemology and has become a sub-discipline^61^. Studies of symbiosis theory in industrial economics have mostly focused on the design of eco-industrial parks^62–64^, lacking regional-scale evaluations. According to Odum^65^’s classification of interspecific interactions, type I corresponds to mutually beneficial symbiotic relationships between species. Ty II and Type IV correspond to partially beneficial symbiotic relationships, and Type III corresponds to mutually competitive relationships between two species. That is to say, the ES and the IS could form symbiotic patterns through positive–negative feedbacks and cause-effects as symbiotic units in the symbiotic system. The application of symbiosis theory in IE research can systematically and comprehensively clarify the nature and process of the interaction between IS optimization and ES enhancement, which is appropriate and reasonable.

### Evaluation model

This paper referred to the study of Sun and Wang^1,47^ to calculate the IES development level and adopted the grey Lotka–Volterra to analyze the relationship between IS and ES, see below:

1 $$ \left\{ {\begin{array}{*{20}l} {\frac{dI}{{dt}} = F_{1} (I,E) = r_{1} I\left( {\frac{{K_{1} - I + \theta_{1} E}}{{K_{1} }}} \right)} \hfill \\ {\frac{dE}{{dt}} = F_{2} (I,E) = r_{2} E\left( {\frac{{K_{2} - E + \theta_{2} I}}{{K_{2} }}} \right)} \hfill \\ \end{array} } \right. $$

where I and E denote the industrial system index (ISI) and ecosystem index (ESI), respectively. $r_{1}$ denotes the growth rate of the system i. $K_{i}$ represents the highest development level of system i, and $\theta_{1}$ and $\theta_{2}$ indicate the impact coefficients of ES on IS and IS on ES, respectively; if $\theta_{i}$ is greater than zero, the impact is the promoting effect; otherwise, the inhibiting effect. The function $f\left( {\theta_{1} ,\theta_{2} } \right)$ was constructed to represent the symbiotic coordination between systems. This paper defined the symbiotic coordination degree (C) as follows: $C = f\left( {\theta_{1} ,\theta_{2} } \right) = \frac{{\theta_{1} + \theta_{2} }}{{\sqrt {\theta_{1}^{2} + \theta_{2}^{2} } }}$, $C \in \left[ { - \sqrt 2 ,\sqrt 2 } \right]$.when the two systems promote each other, $C \in \left[ {1,\sqrt 2 } \right]$; when the two inhibit each other, $C \in \left[ { - \sqrt 2 , - 1} \right]$.when the two are unilaterally inhibited, $C \in \left[ { - 1,1} \right]$.If $\theta_{1} + \theta_{2} \ge 0$,$C \in \left[ {0,1} \right]$, the two systems have some complementarity and could develop into a symbiotic relationship. If $\theta_{1} + \theta_{2} \le 0,\,C \in \left[ { - 1,0} \right]$, one system's impact on another system is too strong, and could transform into a competitive relationship. The larger the C, the more mutually beneficial and symbiotic between the systems, while the smaller the C, the more mutually detrimental and competitive between the systems. Equation (1) can be expressed as follows:

2 $$ \left\{ {\begin{array}{*{20}l} {\frac{dI}{{dt}} = F_{1} (I,E) = X\left( {a_{0} + a_{1} X + a_{2} Y} \right)} \hfill \\ {\frac{dE}{{dt}} = F_{2} (I,E) = Y\left( {b_{0} + b_{1} X + b_{2} Y} \right)} \hfill \\ \end{array} } \right. $$

The data could be discretized using the mapping relation between the grey derivative and the even logarithm in grey theory^43^. *dI*/*dt*, *dE*/*dt* constitutes a mapping relationship to even logarithms $\left( {I_{{\left( {t + 1} \right)}} ,I_{(t)} } \right),\,\left( {E_{(t + 1)} ,E_{(t)} } \right)$ respectively. Taking the background value $\frac{{I_{(t + 1)} + I_{(t)} }}{2},\frac{{E_{(t + 1)} + E_{(t)} }}{2}$ at year t, and Eq. (2) can be expressed as:

3 $$ \left\{ {\begin{array}{*{20}l} {I_{(t + 1)} - I_{(t)} = a_{0} \cdot \frac{{I_{(t + 1)} + I_{(t)} }}{2} + a_{1} \left[ {\frac{{I_{(t + 1)} + I_{(t)} }}{2}} \right]^{2} + a_{2} \frac{{I_{(t + 1)} + I_{(t)} }}{2}\frac{{E_{(t + 1)} + E_{(t)} }}{2}} \hfill \\ {E_{(t + 1)} - E_{(t)} = b_{0} \frac{{E_{(t + 1)} + E_{(t)} }}{2} + b_{1} \left[ {\frac{{E_{(t + 1)} + E_{(t)} }}{2}} \right]^{2} + b_{2} \frac{{E_{(t + 1)} + E_{(t)} }}{2}\frac{{I_{(t + 1)} + I_{(t)} }}{2}} \hfill \\ \end{array} } \right. $$

The parameters can be obtained as follows: ${\text{r}}_{1} = {\text{a}}_{0} ,r_{2} = b_{0} ,K_{1} = - \frac{{a_{0} }}{{a_{1} }},K_{2} = - \frac{{b_{0} }}{{b_{1} }},\theta_{1} = - \frac{{a_{2} }}{{a_{1} }},\theta_{2} = - \frac{{b_{2} }}{{b_{1} }}.$

### The influence mechanism of IE

With the help of Stata software, this paper used the Oprobit model to conduct an empirical analysis of the impact factors of IE. This paper selected the following indicators to study the influence mechanism of IE based on the existing research^2,33,49^. Specifically, the urbanization rate (URB) and GDP per capita (GDP) were chosen to characterize the level of economic development, the interaction of the number of patents granted for inventions (PAT) and R&D expenditure (R&D) was used to represent the level of scientific and technological innovation, and the total energy consumption (RES) and population density (DEN) were selected to indicate ecological pressure. The total amount of import and export, the ratio of the share of tertiary industry to the share of secondary industry, and pollution control investment were used to represent the openness degree (POR), industrial structure (STR), and end-of-pipe control intensity (POL), respectively. The proportion of local fiscal expenditure to GDP (GOV) was used to represent the government's regulatory effort. The higher the degree of marketization, the more private capital enters the industrial market, and enterprises are more motivated to promote innovation, which is conducive to IE. This paper chose the proportion of the number of employees in state-owned economic units to the number of employees in the region to represent the marketization level (MRS). Since the possible kidnapping behavior of state-owned units to local governments could hinder the effect of environmental regulation to some extent, the interaction of MRS and GOV was chosen to characterize the government's influence under marketization^66^. Some variables are logarithmically calculated to reduce the absolute difference between the data. The model could be expressed as follows:

4 $$ \begin{aligned} C = & \beta_{1} {\text{URB + }}\beta_{{2}} {\text{ln\_GDP + }}\beta_{{3}} {\text{DEN + }}\beta_{{4}} \ln \_RES + \beta_{5} \ln \_POR \\ & \quad + \beta_{6} \ln \_POL + \beta_{7} STR + \beta_{8} PAT * R\& D + \beta_{9} MAR * \ln \_GOV + u \\ \end{aligned} $$

### Ethical approval

There is no data collected from human subjects.

## Results

### The IES development level

**Figure 4:**
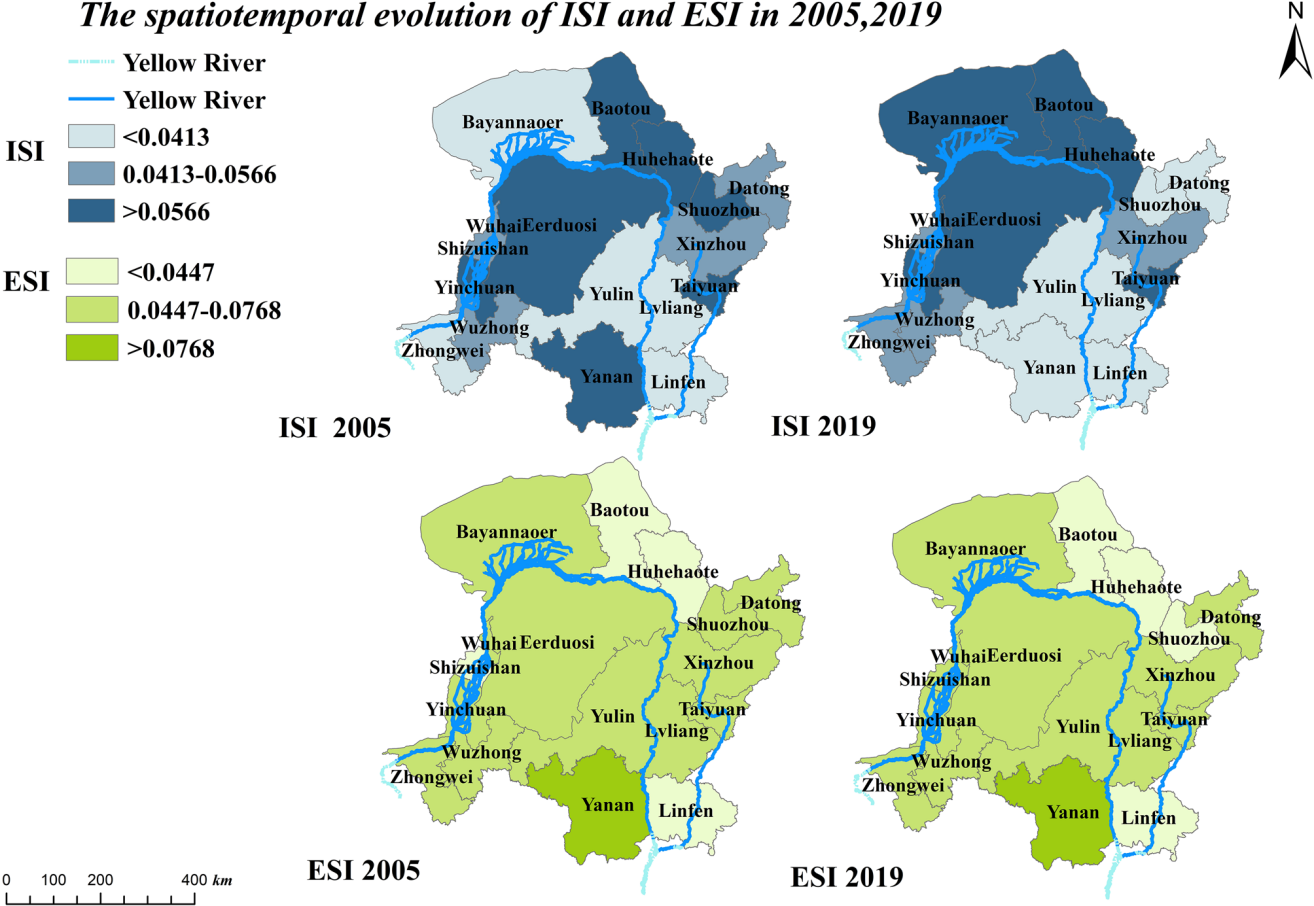
The spatiotemporal evolution of ISI and ESI in 2005 and 2019.

Based on the evaluation index system proposed in the existing research, the IS development index (ISI) and ES development index (ESI) of the 17 cities in the Area from 2005 to 2019 were calculated. The development of IS and ES was unbalanced, with a relatively stable spatial distribution and slight inter-annual variation. High ISI was mainly distributed in the middle reaches of Inner Mongolia. Constrained by poor location conditions and a backward development stage, the ISI level was weak in the upper and lower reaches of the river. Cities with high ESI include Yan'an, Taiyuan, Xinzhou, and Datong, while cities with low ESI include Baotou, Hohhot, and Linfen (Fig. 4).

The extensive mining and processing mode of energy and mineral resources in the Area has aggravated the ecological fragility by changing the geological structure and destroying the surface vegetation. Especially in Yulin, Ordos, Lvliang, Shuozhou, Xinzhou, and Linfen, where the energy sector accounts for more than 70% of the total industrial output^67^. The high value of the ISI index was concentrated in the Hubaoeyu city cluster, which may be attributed to China's National Main Function Zone Plan released in 2011, which listed the cluster as a national critical development zone, promoting the upgrading of advantageous industries and the development of regional integration. The high values of ESI were mainly concentrated in Yanan, Taiyuan, Xinzhou, and Datong. Among them, Yanan has achieved remarkable achievements in ecological construction since the policy of returning farmland to the forest was carried out in 1999, and Taiyuan, as a capital city, has rich resources, superior development conditions, and relatively stable ecological development. Xinzhou and Datong, as mature resource-based cities, are the core areas to safeguard energy and resource security in China at the present stage. Overall, the development of ES and IS was not synchronized, and the different synchronization levels could signify different IE levels.

The Jenks Method is a data classification technique used to classify data based on the natural groupings inherent in the data distribution. ISI and ESI could be divided into three grades using the Jenks method with the help of ArcGIS 10.2 software. This paper defined the development level of IES based on the grades of the ISI and ESI (Table 1). It can be seen that the IES development in most cities of the Curve remained medium-level.

**Table 1:** Classification of the IES.

Development Level	Classification criteria (level 1 is the highest)	City
High-level	Both the ISI and the ESI are at level 1	Taiyuan
Medium-level	Neither the ISI nor the ESI is at level 3 at most one of them is at level 1	Yanan, Xinzhou, Bayannur, Baotou, Ordos, Wuhai, Yinchuan, Lvliang, Linfen, Shuozhou, Datong
Low-level	At least one of the ISI and the ESI is at level 3	Zhongwei, Wuzhong, Yulin, Shizuishan, Hohhot

### The relationship between IS and ES

This paper evaluated the IE-ES symbiotic patterns of the 17 cities in the Curve Area from 2005 to 2019 according to Formula (3). The development of IS and ES in Bayannur, Baotou, Wuhai, Yinchuan, Shuozhou, Taiyuan, and Yan'an promoted each other and were mutually beneficial. The symbiotic relationship in Ordos, Zhongwei, Lvliang, and Xinzhou was the IS beneficial model: if the development focus of these cities was on IS, their ecological environment would be slightly impacted; however, if they focused on ES quality improvement, their IS would also be driven to high-quality transformation. Therefore, the development of such cities should focus on the high-quality transformation of the ecological environment, and drive industrial transformation through symbiotic effects while improving ecological quality. The symbiotic mode of Shizuishan, Datong, and Linfen was the ES beneficial model: the IS development in these cities was conducive to optimizing the ecological environment while focusing on ES improvement would inhibit IS development. Therefore, the development of these cities should focus on the high-quality transformation of the industrial economy, and promote economic growth while driving the construction of ecological civilization through symbiosis.

There were still many cities in the Curve Area that had not escaped the mutual competition of IS and ES, and the symbiotic pattern of Hohhot, Wuzhong, and Yulin was of this type. In this case, increasing the intensity of natural capital utilization would only unilaterally promote IS development and would not form a driving effect on ES quality improvement; increasing investment in ecological environment construction would only unilaterally help ES quality improvement and would not form a positive feedback effect on the industrial economy. In other words, the development of either the industrial economy or the ecological environment alone would result in the slow development of another symbiotic unit. The ES quality is an important basis for realizing the shift of the symbiotic pattern from mutual competition to partially and mutually beneficial symbiosis, therefore, Hohhot, Wuzhong, and Yulin should adhere to the guideline of giving priority to ecology and make further development plans after getting rid of the mutual competition model.

On the whole, the IS development and ES promotion in the Curve Area remained in the stage of isolated development or mutual restriction during the study period. The situation of industrial development at the expense of the ecological environment was common, and the ecological environment failed to effectively support the industrial development of the Area.

### IE level and IE influencing factors

**Figure 5:**
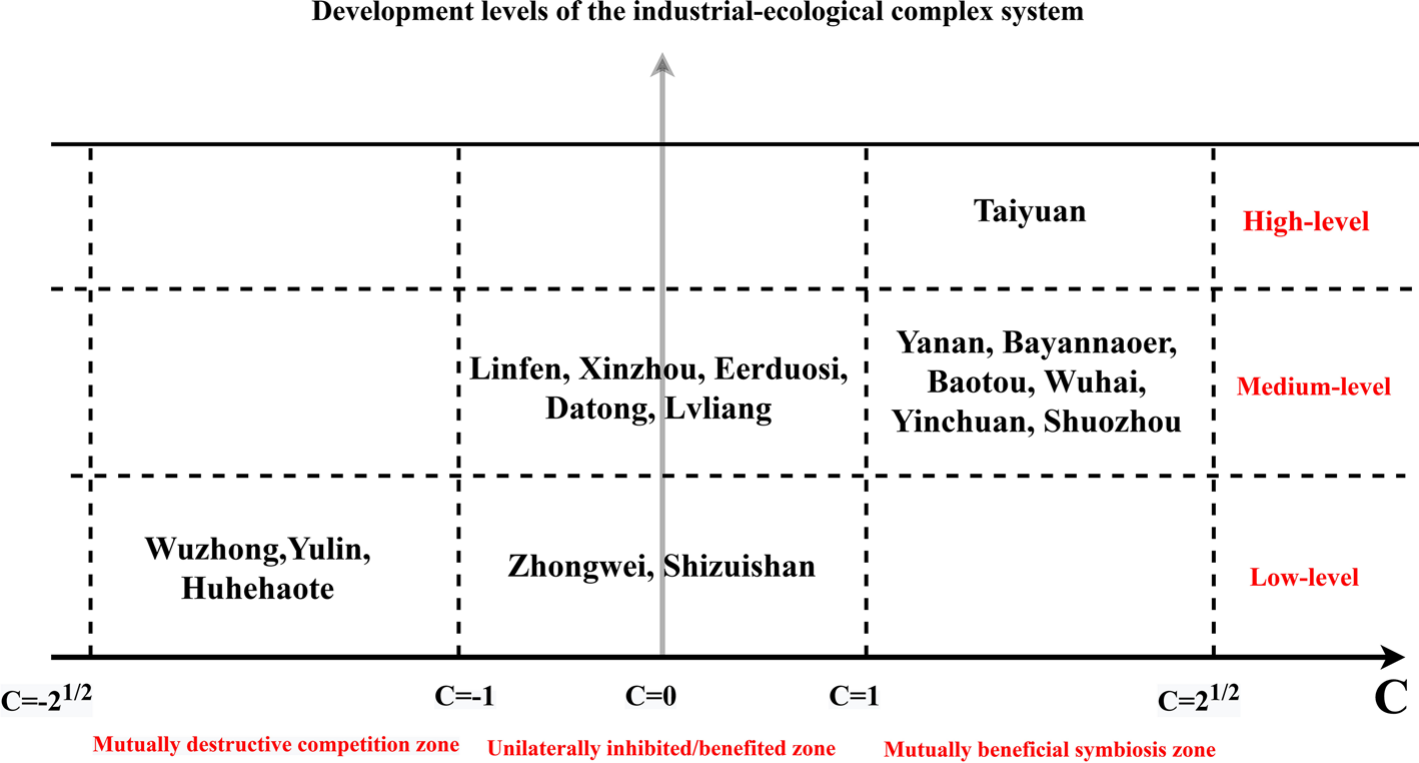
IE level of 17 cities in the Curve Area.

The IE level could be classified based on IES development and IS-ES relation. As shown in Fig. 5, the IE level declined from top to bottom and from right to left. The majority of cities in the Area were in the middle levels.

IE level could be ranked. In this paper, values 1–5 were assigned to cities of low-level mutually inhibited competition type, low-level unilaterally inhibited/benefited type, medium-level unilaterally inhibited/ benefited type, medium-level mutually beneficial symbiosis type, and high-level mutually beneficial symbiosis type, respectively, which characterize the ranking of IE from low to high. The regression results and marginal effects of the impact factors were examined using the Oprobit model with the help of Stata (Table 2).

**Table 2:** The regression results and marginal effects of the impact factors.

Marginal effect								
X	Coef.	Std. Err	Z	1	2	3	4	5
PAT*R&D	0.003*	0.002	1.78	− 0.0002	− 0.0001	− 0.0001	0.0003	0.0001
URB	− 0.180**	0.082	− 2.21	0.0132	0.0067	0.0037	− 0.0177	− 0.0059
ln_GDP	4.305**	1.855	2.32	− 0.3163	− 0.1604	− 0.0880	0.4231	0.1416
DEN	− 0.011	0.009	− 1.20	0.0008	0.0004	0.0002	− 0.0011	− 0.0004
ln_RES	− 5.900**	2.718	− 2.17	0.4336	0.2199	0.1207	− 0.5799	− 0.1941
ln_POR	0.749*	0.433	1.73	− 0.0549	− 0.0279	− 0.0153	0.0735	0.0246
Mar*GOV	− 3.069*	1.728	− 1.78	0.2256	0.1144	0.0628	− 0.3017	− 0.1010
ln_POL	4.358**	1.902	2.29	− 0.3203	− 0.1624	− 0.0891	0.4284	0.1434
STR	6.219**	2.593	2.40	− 0.4568	− 0.2316	− 0.1271	0.6110	0.2045

*Correlation is significant at 10% level.

**Correlation is significant at 5% level.

***Correlation is significant at 1% level.

IE was positively correlated with scientific and technological innovation, economic development level, openness degree, pollution control intensity, and industrial structure. For each unit increase in these indicators, there was a higher probability that IE would move up to a higher level. Urbanization degree, resource consumption, government influence, and IE were negatively correlated. For every unit that the indicators increased, there was a higher probability that IE would fall into a lower tier.

## Discussion

The Area is not only of great significance to safeguarding China's ecological security but also one of the critical areas for China to achieve the goal of being carbon neutral. However, the conflict between the region's environmental protection and economic development is prominent. As a medium connecting economic development and the ecological environment, industrial activities are essential tools for implementing the strategy of ecological protection and high-quality development in the Curve Area. IE, as a powerful development engine under the new normal, can inject new energy into the high-quality development of the Curve Area. The premise of releasing the positive effect of IE is to identify the regional IE level.

Existing studies on IE emphasize the transformation of traditional industrial systems according to the principle of "learning from nature", solving the contradiction between development and protection through the closed-loop cycle of material and energy, so as to achieve the coordinated development of industry and ecology. Therefore, the corresponding research scales are mostly confined to a single small scale in space and time, focusing on the design and construction of eco-industrial parks and the production process of enterprises, ignoring the combination and regional expression of different industrial development patterns. Besides, the transformation of IS by imitating the material and energy flow of the natural ecosystem is to rely on technology to solve the contradiction between development and protection, ignoring the relationship between the industrial organization, industrial layout, industrial structure, and ecological environment and splits the organic link between micro and macro. The few IE studies conducted from a macro perspective mainly focused on the impact of ecological constraints on industrial development and treated the IS as a black box. Resolving the contradictions is not about supporting one party by restricting the development of another party but rather regarding environmental protection and industrial development as a system and pursuing the harmonious coexistence of the two. Due to these limitations, this paper evaluated the IE level according to its definition from a system science perspective: IE is the process of constructing IES with the realization of the mutual benefits for IS and ES, and the IE level could be obtained in a judgment matrix based on the performance of IES and its subsystems. Examining the IE level through the integration of IES quality and IS-ES relation has three significant advantages: First, the IES quality and the symbiotic types reflect different dimensions of the IES level, which facilitates managers and the public to understand and apply the evaluation results thoroughly. Second, the classification of IES quality and symbiotic mode levels can reflect the changes in IE level, which helps to overcome the lag of analysis and achieve the purpose of early warning and guidance. Third, the ES quality determines the upper limit of the IE, and the IS quality determines the lower limit of the IE. Determining the IE level based on the IES quality and IS-ES relations allows scientific measurement and detection of ecological thresholds and development constraints.

Regarding the influencing factors of IE, this study reveals both consistencies and inconsistencies in comparison to previous research. Substantial fiscal revenue serves as the foundational support for the development of IS and ES. The upgrading of industrial structure is conducive to production capacity improvement and ecological optimization, both of which contribute to the evolution of IES. Importantly, industrial upgrading does not necessitate abandoning traditional industries or achieving zero pollution. Instead, it emphasizes the synergistic combination of economic development and ecological preservation. Furthermore, this study highlighted the role of end-of-pipe treatment in promoting IE, which could directly improve environmental quality through pollution control investments and attract social capital for green industries. The study identified a positive correlation between the openness degree and regional IE. The Curve Area has actively engaged in industrial transfers and fostered the modern manufacturing and big data industries, making it an attractive investment destination. This trend was catalyzed by initiatives like the "One Belt, One Road" and "Western Development." Si^68^ contends that West China derives advantages from technology spillover via industrial transfers, which facilitate enhancements in industrial structure and help alleviate the significant risks linked to escalating innovation expenses. As a result, Si posits that in this particular setting, technological innovation may have a detrimental effect on industrial development. Technological innovation and technology diffusion can both improve the output and allocation efficiency of production factors and improve the energy-saving levels of enterprises. The majority of enterprises in the Curve Area lagged behind in terms of production technology, production equipment, and high-end product development. Besides, most of the regions receiving industrial transfers typically serve as refugees from pollution, inheriting predominantly highly polluting heavy industries. Moreover, the demographic dividend that has driven China's rapid economic growth over the past 30 years is about to disappear^69^. That is to say, relying solely on technology spillover is insufficient to realize regional IE instead, technological innovation emerges as the central driver of IE. This clarifies the observed positive correlation between technological innovation and IE. Additionally, FDI often associated with technology transfer and spillover, was also found to promote IE. Notably, the impact of openness degree on IE appears to diminish, while the significance of technological innovation steadily rises. In the post-pandemic era, IS development should prioritize internal circulation, complemented by external circulation, with a proactive focus on enhancing regional technological innovation.

Existing studies generally agree that urbanization has a facilitating effect on the maturation of the IE systems^20,70^. However, urbanization can also bring ecological pressure and resource consumption. An and Li^71^ believe that urbanization in resource-based cities inhibits innovation activities and thus, to a certain extent, IE development. This may explain the negative correlation between urbanization and IE found in this paper. Although the contradiction between supply and demand in the labor market, the disappearance of the demographic dividend, and the transfer of employment in the context of urban–rural integration have led to a weakening of the influence of population density and urbanization level on the IE level, urban expansion at the expense of ecological and environmental quality is still undesirable. Energy consumption is typically seen as having a negative relationship with Industrial Ecology (IE), aligning with our research findings. In areas where resource-based cities are concentrated, the energy and chemical industries stand as the primary sources of carbon emissions and will continue to be essential for economic development in the short term. However, within the context of achieving carbon neutrality, the Curve Area must promptly reconfigure its industrial structure and enhance energy utilization efficiency. Government influence is typically viewed as a positive force for advancing IE^19^, which contradicts the findings of this study. Intense competition among administrative officials for promotions and the prevalence of a GDP-oriented performance appraisal system form the foundation of local government decision-making. Under these institutional arrangements, local governments are highly motivated to prioritize political achievements and economic growth. To maintain economic development and secure upward mobility, local government officials often hesitate to proactively engage in industrial transformation and allocate additional funds for enhancing regional public goods and services. Besides, in regions with inadequate levels of marketization, the presence of state-owned capital can potentially hinder government initiatives. Notably, the marketization level in the four provinces of the Curve Areas falls significantly below the national average level^72^. However, this does not diminish the significance of government in the IE process. In fact, more effective government regulation can reduce obstacles to factor mobility and stimulate greater investment in environmental management. Additionally, the government can foster IE by offering financial incentives to enterprises adopting advanced technologies or achieving lower emissions, while imposing penalties on those with higher emissions to encourage the adoption of cleaner production methods. Consequently, the transformation of the fiscal function and a comprehensive commitment to government regulation are more critical than merely adjusting the scale and structure of fiscal expenditure.

This paper introduces a novel research perspective to industrial ecology, yet there are several limitations to be acknowledged. **Firstly,** as a complex composite system, the study of IE needs a large number of data to corroborate with the actual situation. However, whether it is an IS or an ES, the construction of its evaluation framework can only choose representative indicators. Affected by the availability of data, the appropriate indicators may not be the optimal ones, which may affect the comprehensiveness of the evaluation framework. In the future, with the continuous improvement of statistical data, the development of a single industrial system can be measured, and more targeted development strategies can be proposed accordingly. **Secondly,** the evaluation models used in this paper, such as the LV model, are based on the simulation and simplification of the real situation. Some mathematical estimation methods applied in the model can bias the results, therefore, the improvement of these models and calculation methods in the direction of mathematical economics could be promoted. **Thirdly,** the development suggestions proposed in this paper at the macro level may yield different situations in practical applications due to regional differences and the complex interplay of development factors, plus this paper focused only on the Curve Area without comparisons with other countries or regions. Therefore, further reflection is needed regarding the strategies and recommendations derived from the research findings. In the future, research can examine IE development cases at both macro and micro levels and compare development paths with other countries and regions, so as to further enhance the objectivity and practicality of IE strategies.

## Conclusions

This paper defined IE as the process of constructing industrial-ecological systems (IES) with the realization of the mutual benefits for IS and ES. Therefore, the IE level depends on the development level of the IES and the relationship between IS and ES. Choosing the π-shaped curve area as the study area, this paper referred to the existing research to account for IES development level and adopted the grey Lotka–Volterra to analyze the relationship between IS and ES. The main findings include: The high levels of ISI were mainly distributed in the middle reaches of Inner Mongolia. Cities with high ESI include Yan'an, Taiyuan, Xinzhou, and Datong, while cities with low ESI include Baotou, Hohhot, and Linfen. The development level of the IES in most of the cities in the Area was at medium-level. There are three types of interaction between IS and ES: mutually beneficial symbiosis, partially beneficial symbiosis, and mutual competition. On the whole, the IS development and ES promotion in the Curve Area remained in the stage of isolated development or mutual restriction during the study period. The situation of industrial development at the expense of the ecological environment was common, and the ecological environment failed to effectively support the industrial development of the Area. Based on the IES development and ES-IS interaction, the IE level of each city in the Area could be divided into five levels. Using the Oprobit model, IE was found to be positively correlated with scientific and technological innovation, economic development level, openness degree, pollution control intensity, and industrial structure while negatively correlated with urbanization degree, resource consumption, and government behavior.

Due to the overall fragility of the ES in the Curve Area, existing studies often emphasize environmental protection and resource conservation, while blindly restricting industrial development. However, China's industrial economic development will not be able to get rid of resource dependence for a long time in the future. For the Curve Area, the development goal of IE should not be set on the ecological quality being unaffected, or material reduction or material recycling, but on a higher level to pursue the high-quality development of IS and ES, the mutually beneficial symbiosis between the two, and sustainable regional development. In this process, the roles of government and the market need to be given full play. Especially in the post-epidemic period, the development in the Area especially needs the support of national forces. Only by incorporating ecological capital into rational choices, restraining, and stimulating through institutional means, and adopting economic signals to turn ecological scarcity into economic scarcity, can economic subjects be prompted to make rational decisions through profit and loss opportunities, forming the endogenous driving force for IE. In other words, the IE process requires the synergy of government, society, and market economy (Supplementary Tables 1, 2 and 3).

## Data availability

The datasets used and/or analyzed during the current study are available from the corresponding author on reasonable request.

### Supplementary Information

Supplementary Tables.

## Abbreviations

ES: Ecological system
IS: Industrial system
IES: Industrial-ecological system
IE: Industrial ecology
ESI: Ecological system development index
ISI: Industrial system development index
LV: Lotka–Volterra
GLV: Grey Lotka–Volterra

## References

[1] Sun Y, Wang N (2022). Evolution and obstacle factors of high-quality industrial development in the π-shaped Curve Area of the Yellow River basin in China. J. Geog. Sci..

[2] Sun Y, Wang N (2023). An evaluation framework for industrial ecology based on the symbiosis theory-taking the π-shaped curve area in the Yellow River of China as an example. Ecol. Ind..

[3] Sun Y, Wang N (2021). Development and correlations of the industrial ecology in China’s Loess Plateau: A study based on the coupling coordination model and spatial network effect. Ecol. Ind..

[4] Frosch RA, Gallopoulos NE (1989). Strategies for manufacturing. Sci. Am..

[5] Côté R, Hall J (1995). Industrial parks as ecosystems. J. Clean. Prod..

[6] Graedel TE, Allenby BR (1995). Industrial Ecology.

[7] Gertler, N. *Industry ecosystems: Developing sustainable industrial structures*, Massachusetts Institute of Technology, (1995).

[8] Lambert AJD, Boons FA (2002). Eco-industrial parks: Stimulating sustainable development in mixed industrial parks. Technovation.

[9] Fan H, Cheng Y (2004). Industry ecology: A perspective on enterprise competition. China Ind. Econ..

[10] Erkman S (2001). Industrial ecology: A new perspective on the future of the industrial system. Swiss Med. Wkly..

[11] Wu, J. & Peng, F. Development and realization path of industrial ecology. *Hunan Soc. Sci.*, 149–151 (2013).

[12] Yuan, Z. & Bi, J. Recent research progress and trend outlook of industrial ecology. *J. Ecol.* 2709–2715 (2006).

[13] Kaufman, S. M. in *Metropolitan Sustainability* 40–54 (Woodhead Publishing, 2012).

[14] Bringezu, S. & Schütz, H. in *Eurostat, Theme 2 Economy and Finance* Vol. 2 (Wuppertal: Wuppertal Institute, Luxembourg, 2001).

[15] Dahlström K, Ekins P (2005). Eco-efficiency trends in the UK steel and aluminum industries. J. Ind. Ecol..

[16] Odum HT (1996). Environmental Accounting-Emergy and Environmental Decision Making.

[17] Li, J., Wang, Y. & Zhou, M. in *International Conference on Energy, Environment and Sustainable Development (ICEESD 2011).* 1249–1254 (2012).

[18] Song M, Guan Y, Wang J, Zhao J (2016). Evaluation of urban industrial ecological transformation in China. Clean Technol. Environ. Policy.

[19] Guo F, Tong L, Liu Z, Zhao H, Hou A (2019). Spatiotemporal pattern and influencing factors of industrial ecology in Shandong Province: Based on panel data of 17 cities. Geogr. Res..

[20] Chen Y, Li X, Sun Y, Chen Y (2020). Evolution characteristics and its influencing factors of industrial ecology in the coastal areas of China. Econ. Geogr..

[21] Liu S, Wang L, Yin P, Guo F (2018). Spatiotemporal characteristics and its driving factors of industrial ecology of prefecture-level cities in China. Resour. Dev. Market.

[22] Wang, J. *Analysis of Chaotic Characteristics in the Evolution of Industrial Economic System in Gansu Province* Master thesis, Lanzhou University, (2013).

[23] Guo L (2007). Study on System Dynamics Model of Structure Evolution of the Industrial Economic System.

[24] Fan S (2018). Symbiotic Relationship Between Industrial Structure Optimization and Land use Efficiency Improvement.

[25] Qiu, Y. *Research on the Development of Industrial Ecology Viewed from a Scientific Outlook on Development* Doctor thesis, Hunan University, (2013).

[26] Wang Y (2021). Research on the Security of Regional Innovation Ecosystem.

[27] Zhang X (2020). Research on the Symbiosis of Regional Innovation Ecosystem in China.

[28] Chen, M. in *Proceedings of The 2nd Asia-Pacific Management and Engineering Conference (APME 2016).*

[29] Wu, Q. X. & Ieee. in *International Conference on Robots and Intelligent System (ICRIS).* 413–417 (2019).

[30] Hui, H. & Hao, Y. in *Proceedings of the 2020 International Conference on Social Sciences and Big Data Application (ICSSBDA 2020).* 222–229 (Atlantis Press).

[31] Ren D, Cao G, Long S (2021). Analysis of the coordination degree of social development in China based on the framework of HDI. J. Quant. Technol. Econ..

[32] Tang L, Li J, Yu L, Qin D (2010). Quantitative evaluation methodology for system coordination development based on distance coordination degree model. Syst. Eng. Theory Pract..

[33] Sun Y, Wang N (2021). Eco-efficiency in China’s Loess Plateau Region and its influencing factors: A data envelopment analysis from both static and dynamic perspectives. Environ. Sci. Pollut. Res..

[34] Zhao, J., Tian, L., Bai, Y., He, J. & Li, P. Research on the Coupling of Energy Consumption and High-quality Development in the Yellow River Basin. *E3S Web Conf.* **261**, 01061 (2021).

[35] Yu X, Zhang Y (2021). An economic mechanism of industrial ecology: Theory and evidence. Struct. Chang. Econ. Dyn..

[36] Lotka AJ (1925). Elements of physical biology. Nature.

[37] Volterra V (1926). Variazioni e fluttuazioni del numero d'individui in specie animali conviventi. Memoria della Regia Accademia Nazionale dei Lincei, Series.

[38] Lauro FM (2011). An integrative study of a meromictic lake ecosystem in Antarctica. ISME J..

[39] Szolnoki A (2014). Cyclic dominance in evolutionary games: A review. J. R. Soc. Interface.

[40] Zhang R (2019). An evaluating model for smart growth plan based on BP neural network and set pair analysis. J. Clean. Prod..

[41] Xia M, He X, Zhou Y (2021). Symbiosis evolution of science communication ecosystem based on social media: A Lotka–Volterra model-based simulation. Complexity.

[42] Wu L, Wang Y (2011). Estimation the parameters of Lotka–Volterra model based on grey direct modelling method and its application. Expert Syst. Appl..

[43] Wu L, Liu S, Wang Y (2012). Grey Lotka–Volterra model and its application. Technol. Forecast. Soc. Change.

[44] Mao S, Zhu M, Wang X, Xiao X (2020). Grey-Lotka–Volterra model for the competition and cooperation between third-party online payment systems and online banking in China. Appl. Soft Comput. J..

[45] Zhang Y, Huang G (2021). Grey Lotka–Volterra model for the co-evolution of technological innovation, resource consumption, environmental quality, and high-quality industrial development in Shaanxi Province, China. Environ. Sci. Pollut. Res..

[46] Wang Z, Zhu H (2016). Testing the trade relationships between China, Singapore, Malaysia and Thailand using grey Lotka–Volterra competition model. Kybernetes.

[47] Sun Y, Wang N (2022). Sustainable urban development of the π-shaped Curve Area in the Yellow River basin under ecological constraints: A study based on the improved ecological footprint model. J. Clean. Prod..

[48] Sun Y, Wang N (2022). Evolution and obstacle factors of high-quality industrial development in the π-shaped Curve Area of the Yellow River basin in China. J. Geogr. Sci..

[49] Sun Y, Wang N (2022). Sustainable evaluation of the eco-economic systems in the π-shaped Curve Area of the Yellow River basin of China: A study based on the 3D ecological footprint model. Environ. Sci. Pollut. Res..

[50] National Bureau of Statistics. (China Statistics Press, 2005–2019).

[51] China's Ministry of Housing and Urban-Rural Development. (China Economics Publishing House, 2005–2019).

[52] National Forestry and Grassland Administration. (China Forestry Publishing House, 2005–2019).

[53] Shaanxi Provincial Bureau of Statistics. (China Statistics Press, 2005–2019).

[54] Shanxi Provincial Bureau of Statistics. (China Statistics Press, 2005–2019).

[55] Ningxia Bureau of Statistics. (China Statistics Press, 2005–2019).

[56] Inner Mongolia Bureau of Statistics. (China Statistics Press, 2005–2019).

[57] Bureau of Urban Statistics. (China Statistics Press, 2005–2019).

[58] Paracer S, Ahmadjian V (1988). Symbiosis: An Introduction to Biological Associations.

[59] Bian C (2008). Fusion and Symbiosis: Japanese Philosophy from the Perspective of East Asia.

[60] Chunqing Y (2002). Financial Symbiosis Theory and Urban Commercial Bank Reform.

[61] He, Z. & Xu, J. Summary of the development of biological symbiosis theory and its application in other fields. *Entrepreneur World (Theory Edition)*, 138–141 (2006).

[62] Chen F (2020). Research on Planning, Design, and Evaluation of Ecological Industrial Parks under the Concept of Cycle Symbiosis.

[63] Zhao, H., Xu, Z. & Chen, R. The behavior pattern of eco-intelligent enterprise symbionts and their symbiosis economic benefits. *Chinese Manag. Sci.*, 131–137. 10.16381/j.cnki.issn1003-207x.2004.06.024 (2004).

[64] Zhang Y (2009). EIPs industrial symbiosis system construction and Nodal relationship. J. Wuhan Univ. Technol..

[65] Odum EP (2009). Principles of Ecology.

[66] Chen L, Kong F, Wen C, Wang Y (2021). Analysis of regional differences and influencing factors of eco-efficiency of river basin industry: A case study of Jinsha river. J. Chongqing Technol. Bus. Univ. (Social Sciences Edition).

[67] Wei, S. Ecological security, heavy chemical industry upgrade, and smart green development in the middle Yellow River. *Econ. Forum* (2020).

[68] Si, L. Spatiotemporal evolution and driving mechanism of industrial ecology in western China. *Gansu Soc. Sci.*, 149–156. 10.15891/j.cnki.cn62-1093/c.2021.04.019 (2021).

[69] Cai F (2012). Demographic transition and sustainable development in China. Proc. Chin. Acad. Sci..

[70] Wang L, Gong X (2014). Urbanization, industrial ecology, and economic growth-An empirical research based on Northwestern five provinces' panel data. China Sci. Technol. Forum.

[71] An S, Li R (2020). Intension and promotion strategy of high-quality development in the Yellow River basin. Reform.

[72] Wang, X., Fan, G. & Hu, L. Marketization Index of China’s Provinces: Neri Report 2018. 739–746 (Beijing, 2019).

