# Supplementary material for: Evaluation of industrial ecology in the π-shaped curve area of China’s Yellow River based on the grey Lotka–Volterra model
Source: Sci Rep. 2023 Nov 4;13:19089. doi: 10.1038/s41598-023-46618-7 (PMC10625620; doi:10.1038/s41598-023-46618-7)
Supplement: Supplementary file 1 — Supplementary Tables. [file 41598_2023_46618_MOESM1_ESM.docx]

**Table 1 The indicator system for calculating ISI and ESI**

| System | Category | Sub-category | Explanations | Variables |
| --- | --- | --- | --- | --- |
| IS | Development momentum | Rooted force |  | The GDP growth rate and per capita GDP |
|  |  | Innovation force | Technological innovation | The proportion of R&D investment in GDP  The ratio of foreign direct investment (FDI) to the total social fixed assets investment |
|  |  |  | Institutional innovation | wages excluding the proportion from State-owned units and urban collective units to total wages |
|  | Development efficiency | Single-factor productivity | Labor productivity | The ratio of GDP to the number of employees in society |
|  |  |  | Capital productivity | The ratio of GDP to the amount of fixed asset investment (stock) in society |
|  |  |  | Energy efficiency | The ratio of GDP to energy consumption |
|  |  | Total factor productivity |  | The Malmquist index |
|  | Development quality | Industrial structure | Structure rationality | The ratio of the tertiary industry to the secondary industry |
|  |  |  | Structure upgrading | The Theil index |
|  |  | Industrial layout | Specialization level | The Locational Quotient (LQ) of manufacturing industries |
|  |  |  | Diversification level | The diversification index |
|  |  |  | Locational degree | The industrial location index |
|  | Development environment | Stable economic environment |  | The growth degree of the current year’s GDP growth rate relative to the previous year’s GDP growth rate, the Consumer Price Index, and the unemployment rate |
|  |  | Harmonious social environment | Development welfare | The number of years of education per capita, the number of medical technicians per 10,000 population, and the road mileage per capita |
|  |  |  | Achievement sharing | The Gini coefficient of GDP and the ratio of urban and rural residents’ income |
| ES | Quality | Evaluation of ecological supply and demand | The quantity comparison between ecological supply and demand | Ecological surplus or ecological deficit |
|  | Efficiency | Evaluation of ecological well-being performance | Human well-being generated by consuming unit natural resources. | Ecological well-being performance |
|  | Structure | Evaluation of ecological supply stability | The equilibrium of ecological carrying capacity | Ecological carrying structure |

**Table 2 The ISI in theπ-shaped Curve Area from 2005 to 2019**

| City | 2005 | 2006 | 2007 | 2008 | 2009 | 2010 | 2011 | 2012 | 2013 | 2014 | 2015 | 2016 | 2017 | 2018 | 2019 |
| --- | --- | --- | --- | --- | --- | --- | --- | --- | --- | --- | --- | --- | --- | --- | --- |
| Bayannur | 0.0402 | 0.0487 | 0.0414 | 0.0457 | 0.0469 | 0.0374 | 0.0437 | 0.0578 | 0.0380 | 0.0321 | 0.0327 | 0.0375 | 0.0516 | 0.0536 | 0.0746 |
| Baotou | 0.0888 | 0.0843 | 0.0684 | 0.0890 | 0.0973 | 0.0968 | 0.0941 | 0.0991 | 0.0972 | 0.1100 | 0.1014 | 0.0975 | 0.0947 | 0.0812 | 0.0953 |
| Ordos | 0.0888 | 0.0908 | 0.0716 | 0.0942 | 0.0982 | 0.1038 | 0.1005 | 0.1084 | 0.0935 | 0.0979 | 0.0960 | 0.1102 | 0.1401 | 0.1261 | 0.1167 |
| Hohhot | 0.0787 | 0.0746 | 0.0654 | 0.0799 | 0.0846 | 0.0847 | 0.0831 | 0.0690 | 0.0756 | 0.0836 | 0.0831 | 0.0887 | 0.0903 | 0.0698 | 0.0849 |
| Wuhai | 0.0535 | 0.0556 | 0.0464 | 0.0556 | 0.0561 | 0.0502 | 0.0557 | 0.0614 | 0.0580 | 0.0593 | 0.0762 | 0.0639 | 0.0666 | 0.0626 | 0.0609 |
| Shizuishan | 0.0485 | 0.0514 | 0.0409 | 0.0510 | 0.0569 | 0.0541 | 0.0476 | 0.0480 | 0.0585 | 0.0653 | 0.0596 | 0.0510 | 0.0555 | 0.0518 | 0.0531 |
| Wuzhong | 0.0447 | 0.0426 | 0.0375 | 0.0373 | 0.0418 | 0.0388 | 0.0389 | 0.0340 | 0.0459 | 0.0460 | 0.0405 | 0.0375 | 0.0421 | 0.0382 | 0.0530 |
| Yinchuan | 0.0679 | 0.0648 | 0.0796 | 0.0628 | 0.0632 | 0.0588 | 0.0623 | 0.0468 | 0.0592 | 0.0620 | 0.0588 | 0.0579 | 0.0630 | 0.0592 | 0.0696 |
| Zhongwei | 0.0376 | 0.0430 | 0.0289 | 0.0402 | 0.0430 | 0.0374 | 0.0373 | 0.0246 | 0.0423 | 0.0427 | 0.0371 | 0.0329 | 0.0292 | 0.0432 | 0.0427 |
| Datong | 0.0566 | 0.0521 | 0.0422 | 0.0514 | 0.0491 | 0.0507 | 0.0515 | 0.0591 | 0.0568 | 0.0549 | 0.0575 | 0.0591 | 0.0503 | 0.0440 | 0.0363 |
| Linfen | 0.0413 | 0.0430 | 0.0983 | 0.0522 | 0.0478 | 0.0437 | 0.0472 | 0.0461 | 0.0463 | 0.0393 | 0.0448 | 0.0415 | 0.0398 | 0.0437 | 0.0316 |
| Lvliang | 0.0274 | 0.0386 | 0.0668 | 0.0411 | 0.0364 | 0.0592 | 0.0538 | 0.0633 | 0.0520 | 0.0286 | 0.0341 | 0.0486 | 0.0341 | 0.0589 | 0.0271 |
| Shuozhou | 0.0745 | 0.0586 | 0.1088 | 0.0580 | 0.0502 | 0.0649 | 0.0568 | 0.0625 | 0.0543 | 0.0436 | 0.0513 | 0.0564 | 0.0372 | 0.0513 | 0.0385 |
| Taiyuan | 0.0886 | 0.0878 | 0.0806 | 0.0920 | 0.1004 | 0.0971 | 0.1014 | 0.0989 | 0.0924 | 0.1027 | 0.0956 | 0.0971 | 0.0941 | 0.0817 | 0.0961 |
| Xinzhou | 0.0500 | 0.0512 | 0.0355 | 0.0474 | 0.0458 | 0.0444 | 0.0467 | 0.0347 | 0.0527 | 0.0554 | 0.0481 | 0.0408 | 0.0388 | 0.0564 | 0.0430 |
| Yanan | 0.0737 | 0.0729 | 0.0573 | 0.0647 | 0.0462 | 0.0442 | 0.0452 | 0.0477 | 0.0373 | 0.0347 | 0.0395 | 0.0424 | 0.0336 | 0.0372 | 0.0381 |
| Yulin | 0.0394 | 0.0401 | 0.0306 | 0.0375 | 0.0361 | 0.0338 | 0.0342 | 0.0386 | 0.0400 | 0.0420 | 0.0438 | 0.0371 | 0.0391 | 0.0410 | 0.0385 |

**Table 3 The ESI in theπ-shaped Curve Area from 2005 to 2019**

| City | 2005 | 2006 | 2007 | 2008 | 2009 | 2010 | 2011 | 2012 | 2013 | 2014 | 2015 | 2016 | 2017 | 2018 | 2019 |
| --- | --- | --- | --- | --- | --- | --- | --- | --- | --- | --- | --- | --- | --- | --- | --- |
| Bayannur | 0.0521 | 0.0517 | 0.0534 | 0.0525 | 0.0484 | 0.0498 | 0.0506 | 0.0563 | 0.0523 | 0.0515 | 0.0525 | 0.0522 | 0.0511 | 0.0558 | 0.0531 |
| Baotou | 0.0447 | 0.0461 | 0.0458 | 0.0448 | 0.0462 | 0.0465 | 0.0431 | 0.0471 | 0.0452 | 0.046 | 0.0467 | 0.0499 | 0.044 | 0.0444 | 0.044 |
| Ordos | 0.0604 | 0.0642 | 0.0667 | 0.0638 | 0.067 | 0.0655 | 0.0661 | 0.066 | 0.068 | 0.0692 | 0.0666 | 0.0709 | 0.0713 | 0.0691 | 0.0667 |
| Hohhot | 0.0192 | 0.0198 | 0.0119 | 0.0145 | 0.0134 | 0.0146 | 0.0127 | 0.0161 | 0.0157 | 0.0135 | 0.0117 | 0.0197 | 0.0115 | 0.0132 | 0.0166 |
| Wuhai | 0.0587 | 0.0583 | 0.0597 | 0.0624 | 0.0631 | 0.0625 | 0.0616 | 0.0617 | 0.0615 | 0.0636 | 0.0662 | 0.0679 | 0.0625 | 0.0663 | 0.0669 |
| Shizuishan | 0.0346 | 0.0337 | 0.0359 | 0.0372 | 0.0358 | 0.0369 | 0.0391 | 0.0423 | 0.041 | 0.0421 | 0.0427 | 0.0441 | 0.0429 | 0.0458 | 0.0454 |
| Wuzhong | 0.0543 | 0.0585 | 0.0541 | 0.059 | 0.0521 | 0.0528 | 0.0567 | 0.0583 | 0.0588 | 0.0582 | 0.0669 | 0.0618 | 0.0643 | 0.0664 | 0.0675 |
| Yinchuan | 0.0656 | 0.0668 | 0.0667 | 0.0667 | 0.0605 | 0.0634 | 0.0618 | 0.0568 | 0.0522 | 0.0512 | 0.0497 | 0.0495 | 0.0458 | 0.0439 | 0.0451 |
| Zhongwei | 0.0697 | 0.0634 | 0.0654 | 0.0649 | 0.0612 | 0.0597 | 0.0581 | 0.0611 | 0.059 | 0.0577 | 0.0661 | 0.0615 | 0.0644 | 0.0662 | 0.0672 |
| Datong | 0.0649 | 0.0660 | 0.0663 | 0.0686 | 0.0711 | 0.0754 | 0.068 | 0.0728 | 0.0701 | 0.0649 | 0.0615 | 0.0607 | 0.0616 | 0.0611 | 0.0611 |
| Linfen | 0.0434 | 0.0451 | 0.0447 | 0.0427 | 0.0485 | 0.045 | 0.049 | 0.0451 | 0.0498 | 0.0513 | 0.042 | 0.042 | 0.0451 | 0.0404 | 0.0402 |
| Lvliang | 0.0577 | 0.0572 | 0.0583 | 0.0573 | 0.0628 | 0.0593 | 0.0607 | 0.0586 | 0.0605 | 0.0635 | 0.0622 | 0.0581 | 0.0622 | 0.0604 | 0.0601 |
| Shuozhou | 0.0591 | 0.0515 | 0.053 | 0.056 | 0.0485 | 0.0487 | 0.0459 | 0.0458 | 0.0467 | 0.0371 | 0.0345 | 0.0377 | 0.0386 | 0.0409 | 0.0426 |
| Taiyuan | 0.0683 | 0.0717 | 0.0718 | 0.0727 | 0.082 | 0.0812 | 0.0849 | 0.0825 | 0.0844 | 0.0884 | 0.0814 | 0.0815 | 0.0788 | 0.0753 | 0.0733 |
| Xinzhou | 0.0768 | 0.0760 | 0.076 | 0.0784 | 0.0825 | 0.0823 | 0.0812 | 0.0783 | 0.0795 | 0.0808 | 0.0817 | 0.0727 | 0.0746 | 0.073 | 0.0722 |
| Yanan | 0.1104 | 0.1086 | 0.1092 | 0.1024 | 0.0948 | 0.0968 | 0.1009 | 0.0944 | 0.0968 | 0.1016 | 0.1104 | 0.1104 | 0.1223 | 0.1217 | 0.1212 |
| Yulin | 0.0601 | 0.0612 | 0.0609 | 0.0561 | 0.0621 | 0.0597 | 0.0596 | 0.0569 | 0.0585 | 0.0593 | 0.0574 | 0.0594 | 0.0592 | 0.0561 | 0.0568 |
